# Facial injuries from bear attacks: A retrospective analysis highlighting periorbital trauma and the need for tissue defect management

**DOI:** 10.1016/j.jpra.2026.09.029

**Published:** 2026-09-30

**Authors:** H. Wakai, A. Onodera, I. Sone, S. Azuma, T. Honda, M. Sakuraba

**Affiliations:** a Department of Plastic and Reconstructive Surgery, Iwate Medical University, 1-1-1 Idaidori, Yahaba-cho, Shiwa-gun, Iwate Prefecture, 028-3694, Japan; b Department of Plastic, Reconstructive and Aesthetic Surgery, Nippon Medical School Hospital, 1-1-5 Sendagi, Bunkyo-ku, Tokyo, 113-8603, Japan

**Keywords:** Bear injuries, Reconstructive surgical procedures, Facial injuries, Wounds and injuries, Traumatology

## Abstract

**Objective:**

Bear attacks often cause severe facial trauma requiring complex surgical management. We aim to analyze facial injuries caused by *Ursus thibetanus* and discuss optimal treatment.

**Methods:**

We conducted a single-center retrospective study of patients with facial trauma caused by bear attacks who were treated at Iwate Medical University Hospital (2017–2022). We analyzed demographic information, injury details (skin, soft tissue, bone involvement), treatment approaches, number of surgeries, and complications. Associations between Facial Injury Severity Scale (FISS) scores, tissue defects, infection, and length of hospital stay (LOS) were evaluated using Spearman's rank correlation coefficient, Mann–Whitney U test, and multivariable regression analysis. *p*< 0.05 was considered statistically significant.

**Results:**

A total of 26 patients (22 men, 4 women; median age, 70.5 years [interquartile range (IQR), 66.3–76.5]) sustained facial skin injuries; the eyelid was the most affected site (20 patients). Soft tissue defects were observed in 9 patients and facial fractures in 16 patients. Critical structures (globe, lacrimal canaliculus, parotid duct, facial nerve) were damaged in 14 patients, with 5 globe ruptures. The median FISS was 2.00 [IQR, 1.00–3.00]. Infection occurred in 9 patients. Overall median LOS was 15.0 days [IQR, 9.25– 28.8]. Patients with tissue loss had significantly longer LOS (median, 30.0 days [IQR, 28.0–48.0] vs. 11.0 days [IQR, 8.0–15.0]; *p* < 0.001). Neither infection nor FISS significantly affected LOS (*p* = 0.152 and *p* = 0.820, respectively).

**Conclusions:**

Bear attacks frequently cause severe facial injuries, often affecting the eyes. Tissue loss significantly prolongs LOS.

## Introduction

Bear-human interactions around the world result in human casualties.^1^^,^^2^ In 2024, the Japanese government officially reported 210 incidents from *Ursus thibetanus (U. thibetanus),* the highest rate seen for a decade.^3^ Most bear injuries involve the face and head.^4^^,^^5^ In Japan, two bear species are resident: the brown bear (*Ursus arctos*) and the Asian black bear (*Ursus thibetanus*). Brown bears inhabit only Hokkaido, whereas Asian black bears are distributed throughout Honshu and the islands to the south.^6^^,^^7^ Consequently, all bear-related injuries observed at our institution are attributable exclusively to Asian black bears. Fortunately, *U. thibetanus* injuries are rarely fatal: prior reports describe mortality rates of 0% to 2.39%.^4^^,^^8^ However, the wounds from bear attacks, especially around the face, are very complex and tend to require multiple surgeries and prolonged hospitalization. The goal of treatment is to obtain a good aesthetic result and return the patient to daily life. Some facial injuries caused by bears require extensive surgery, including tissue transplantation. We report a series of bear-attack injuries, focused on facial trauma, and we discuss treatment for these injuries.

## Methods

We conducted a single-center retrospective study of all patients admitted with facial trauma caused by *U. thibetanus t*o the Department of Plastic and Reconstructive Surgery at Iwate Medical University Hospital between January 2017 and December 2022. The institutional review board granted approval for this study (MH2024-017), and patients were able to opt out of consent for research participation. This study was conducted in accordance with the STROBE guidelines.^9^

On presentation, each patient was evaluated to assess their airway and circulation. We then examined each patient and documented the types of injuries (ie, skin, soft tissue, bone) through careful physical and pertinent radiographic examination. The antimicrobial agents were selected based on the review of the bacteriology of bear bite wounds.^1^ A β-lactam / β-lactamase inhibitor was administered as first-line therapy. Patients with severe contamination or a drug allergy received alternative or additional coverage at the physician’s discretion.

On chart review, we collected demographic data, including age and sex, and details from the medical records regarding the injury sites and treatments of maxillofacial fractures, skin/soft tissue, length of hospital stay (LOS) (days), duration of antibiotics (days), the number of surgeries performed, Facial Injury Severity Scale (FISS) scores.^10^ and any complications (such as infection) during the initial hospitalization. FISS scores were calculated from clinical records and computed tomography (CT) data. When laceration length was unavailable, this component was excluded from the calculation. For comminuted fractures not classifiable as typical Le Fort, naso-orbital-ethmoid (NOE), or zygomaticomaxillary complex (ZMC) patterns, FISS scores were calculated based on individual fracture sites identified on CT. Continuous variables are presented as medians and interquartile ranges (IQRs), and categorical variables as frequencies and percentages. Associations between FISS scores and LOS were assessed using Spearman’s rank correlation coefficient. Differences in LOS according to the presence or absence of soft tissue defects or infection were assessed using the Mann–Whitney U test. Multivariable regression analysis was performed to explore factors associated with LOS (including FISS score, soft tissue defects, and infection). A Generalized Linear Model (GLM) with a gamma distribution and log link function was used because LOS was right-skewed (Supplemental Figure 1). All tests were two-sided, and statistical significance was set at *α*=.05. R version 4.5.1 (packages: tableone, dplyr, broom) was used for all analyses.

## Results

The study sample consisted of 26 patients (22 male patients and 4 female patients) with facial injuries caused by bear mauling. The median age was 70.5 years [IQR, 66.3–76.5]. All patients presented for medical treatment within 24 hours of the bear attack. There was no mortality during the initial presentation period. The median FISS was 2.00 [IQR, 1.00–3.00] (Table 1 and Supplemental Figure 1).

**Table 1:** Demographic and clinical characteristics of the 26 patients (median [IQR] or n (%)).

Variable	Overall (n=26)
Age (median [IQR])	70.5 [66.25-76.50]
Sex Men (%)	22 (84.6)
LOS (days) (median [IQR])	15.0 [9.25-28.75]
Number of surgeries (median [IQR])	1.00 [1.00-2.00]
FISS score (median [IQR])	2.00 [1.00-3.00]

Abbreviations: IQR, interquartile range; LOS, Length of hospital stay; FISS, Facial Injury Severity Scale.

### Details of Skin, bone, and important organ injuries on the face

All 26 patients had facial skin injuries. The eyelid was the most frequently damaged site, with involvement noted in 20 patients (77%). Wounds were described in the cheek area of 19 patients (73%) and the forehead area of 17 patients (65%). Nine patients (35%) had facial soft tissue defects, and 16 patients (62%) had facial-bone fractures. The nasal-ethmoid region was the most frequently fractured (10 patients), followed by the zygoma region in 8 patients and the maxilla region in 6 patients (Table 2). Important organs, including the globe, lacrimal canaliculus, parotid duct, and facial nerve, were injured in 14 patients (54%).

**Table 2:** Details of Facial Injuries From Bear Attacks.

Injury Assessment	n	Percentage
**Regions of lactation on Face**		
Eyelid	20	77%
Cheek	19	73%
Forehead	17	65%
Lip	9	35%
Nose	8	31%
Ear	6	23%
Chin	4	15%
Soft Tissue defect on face (Yes)	9	35%
**Facial Bones Fractures**	**16**	**62%**
Nasal / Naso-ethmoid area	10	38%
Zygoma	8	38%
Maxilla	6	23%
Forehead	3	12%
Mandible	2	8%
Orbital wall	1	4%
**Significant Facial Injuries**	**14**	**54%**
Lacrimal canaliculus	8	31%
Globe	5	19%
Facial nerve	3	12%
Parotid duct	2	8%

Percentages were calculated based on the total number of patients (n = 26). Lip: Upper lip in 7 patients, lower lip in 3 patients.

### Therapeutic measures

Most of the primary surgeries were performed under general anesthesia. All wounds were aggressively irrigated and debrided. For the clinically non-contaminated wounds without extensive tissue defects, we performed primary closure on the day of presentation, using sutures and drainage tubes as appropriate(Table 3). Twenty-five patients received anti-tetanus human immunoglobulin and 8 received the tetanus toxoid vaccine. All patients received prophylactic antibiotics for a median of 7.50 days [IQR, 5.00–9.00]: 5 patients received sulbactam sodium/ampicillin sodium monotherapy, 14 received piperacillin/tazobactam monotherapy, and the others received cefazolin sodium, levofloxacin, piperacillin sodium, or multiple drugs. Some patients whose soft tissue defects or mandible fractures could not be fully treated by primary surgery underwent secondary surgeries(Table 3 and Supplemental Table 1).

**Table 3:** Patient demographics, facial soft tissue injury characteristics, and management.

Case	Age/ Sex	Soft Tissue Defect (Size in cm)	Associated Facial Organ Injuries	Primary Management	Additional Treatment
1	60 / M	—	—	SC	—
2	75 / M	—	L-PD	SC, PD repair	—
3	70 / M	—	L-CL	SC, Canalicular repair	—
4	77 / M	—	—	SC	Scar contracture release
5	62 / M	R-Forehead (3 × 3)	—	SC, Skin graft	—
6	71 / F	R-Cheek (3 × 2)	R-FN	SC, Skin graft	—
7	74 / M	Nose, R-Eyelid, Forehead (N/A)	R-CL, R-GR, R-PD, R-FN, CSF leak	SC Enucleation, CSF leak repair	Free flap (ALT)
8	67 / M	L-Forehead, L-Eyelid (10 × 10)	L-GR	SC, Enucleation, ALT free flap	2nd: Re-anastomosis (venous thrombosis); 3rd: flap removal; 4th: STSG (due to necrosis)
9	69 / M	—	L-GR	SC, Conjunctival suture	2nd: R-lower eyelid ectropion repair; 3rd: Dacryocystorhinostomy
10	69 / M	—	CL	SC, Canalicular repair	—
11	67 / F	L-Ear (3 × 1.5, 3 × 2.5)	—	SC, Artificial dermis	Skin graft
12	83 / M	—	R and L-CL	SC, Canalicular repair	Frontalis suspension (Gore-Tex) for ptosis
13	64 / M	—	Supraorbital nerve	SC, Nerve suture	—
14	66 / M	—	—	SC	—
15	45 / M	—	R-CL, L-FN	SC	—
16	82 / M	—	—	SC	—
17	52 / M	—	—	SC	—
18	81 / M	—	R and L-GR	SC, Enucleation	—
19	72 / F	Nose, Cheek, Upper-lip (8 × 10)	—	SC, Artificial dermis	2nd–7th: Staged nasal reconstruction (free ALT flap, forehead/nasolabial flaps, iliac bone graft, STSG, flap delay & division); 8th: Sequestrectomy for bone graft necrosis
20	79 / F	—a	—	SC, Skin graft^a^	—
21	81 / M	Cheek (4 × 2); Ear (2 × 2)	—	SC, Artificial dermis	Eyelid V-Y flap; ear STSG; nasal reconstruction (nasolabial flap, auricular/costal cartilage grafts)
22	71 / M	—	L-GR	SC, Enucleation	—
23	70 / M	—	L-CL	SC	—
24	65 / M	R-Eyelid & Lower lip (3 × 3); L-Eyelid & Cheek (8 × 10)	—	SC, Artificial dermis	2^nd^: ALT free flap; 3^rd^: Lower eyelid ectropion repair (fascia graft)
25	79 / M	Cheek (5 × 1.5)	L-CL	SC, Artificial dermis	Skin graft
26	72 / M	—	L-CL, L-Inferior oblique muscle	SC, Canalicular repair, Inferior oblique muscle suture	—

a Skin defect was present on the arm (upper extremity) only, with no facial defect. Abbreviations: L, left; R, right; SC, Simple closure; CL, Canaliculus; SG, Skin graft; FN, Facial nerve injury; GR, Groebe rupture; PD, Parotid duct; CSF, Cerebrospinal fluid; STSG; Split-thickness skin graft; ALT, Anterolateral thigh

Of the 16 patients with facial fractures, 8 underwent Open Reduction and Internal Fixation (ORIF). Closed reduction (CR) was mainly used for nasal fractures, and one zygomatic fracture required open reduction without fixation. Due to multiple fracture sites, treatments were often combined—including ORIF, CR, and conservative care—while some were managed with observation alone. Additionally, two patients with mandibular fractures underwent delayed ORIF after primary care (Supplemental Table 1). Of the 9 patients with soft tissue defects, 3 underwent primary reconstruction (2 skin grafts, 1 free flap), and the other 6 underwent secondary or multi-stage reconstruction (free flaps, skin grafts, local flaps, etc.) (Table 3). Important organs: Lacrimal canalicular injuries were observed in 8 patients, and lacrimal canalicular repair was possible in 4. Globe rupture occurred in 5 patients (1 bilateral, 4 unilateral). Four underwent eye enucleation, and the other was managed conservatively. All patients with globe rupture lost vision in the affected side. Parotid duct injury was observed in 2 patients. One underwent parotid duct repair, and the other underwent ligation; neither patient experienced a postoperative complication. Nerve repair could not be performed in all three patients with facial nerve injury (Table 2, Table 3).

A total of 20 patients had minor lacerations affecting the upper limbs, and 7 patients had lacerations involving the lower limbs. Primary closure of all such injuries was performed on the day of the injury after thorough irrigation. Penrose drains were inserted as necessary.

### Progress and complications after initial treatment

6 patients (23%) developed a primary infection after their initial surgery. Three had infection in the craniofacial region, and the other 3 had infections of the extremities. Additionally, 3 patients (11%) developed infections at the donor or reconstruction sites, bringing the total to 9 patients who experienced infections during hospitalization. (Supplementary Table 2). Wound cultures grew Candida, Escherichia coli, Enterobacter aerogenes, and Corynebacterium species, among others. All nine patients with infection, including primary, donor, or reconstruction site infections, received antibiotic treatment. Three of them required surgical procedures such as drainage, debridement, and/or irrigation. All patients improved without further intervention.

### Association of Length of Stay with FISS, Soft Tissue Defects, and Infection

The median LOS was 15.0 days [IQR, 9.25– 28.8] (Table 1). Although patients with higher FISS scores tended to have longer lengths of stay, the association did not reach statistical significance (Spearman’s ρ = 0.347, *p* = 0.0820). Patients with soft tissue defects had a significant difference in LOS compared with those without soft tissue defects (median, 30.0 days [IQR, 28.0–48.0] vs. 11.0 days [IQR, 8.0–15.0]; *p* < 0.001). The infection was not associated with LOS (*p* = 0.152) (Table 4). In the exploratory analysis, a GLM was performed to examine the factors associated with LOS (n = 26). Multivariable analysis revealed that the presence of facial soft tissue defect was independently associated with a significantly longer LOS (Adjusted Ratio = 2.50, 95% CI: 1.63–3.88, *p* < 0.001). In contrast, the presence of infection (Adjusted Ratio = 1.07, 95% CI: 0.688–1.68, *p* = 0.776) and FISS scores (Adjusted Ratio = 1.09 per 1-point increase, 95% CI: 0.946–1.26, *p* = 0.215) showed no statistically significant association with the LOS for other variables.

**Table 4:** Comparison of length of hospital stay according to soft tissue defect and infection status.

Factor	No. of Patients (n = 26)	LOS (days), Median [IQR]	*p*-value
**Soft Tissue Defect**			
Present	9	30.0 [28.0–48.0]	< 0.001*
Absent	17	11.0 [8.00–15.0]	
**Any Infection**			
Present	9	22.0 [14.0–35.0]	**0.152**
Absent	17	14.0 [9.00–21.0]	

Standard comparisons were performed using the Mann-Whitney U test. *p*< 0.05 was considered statistically significant. Abbreviations: IQR, interquartile range; LOS, Length of hospital stay.

### Representative case (Case 24 in Table 3)

Because this case demonstrates a typical pattern of periorbital and midfacial trauma requiring staged reconstruction, it was selected as a representative example.

A 65-year-old man encountered a bear while collecting bamboo shoots and sustained injuries to his face and left little finger. He was airlifted to our tertiary emergency center, where he was found to be alert with stable vital signs. Examination revealed deep wounds and soft tissue loss involving an area of 8 × 10 cm including the left lower eyelid and left cheek; the injury exposed the orbital fat and prevented eyelid closure (Fig. 1A), but there was no damage to the medial canthal ligament or the lacrimal canaliculi, and visual acuity and ocular motility were intact. Additionally, a 3 × 3 cm skin defect at the right corner of the mouth involved part of the orbicularis oris muscle. The left little finger sustained a laceration, but movement was intact and there was no fracture evident on X-ray. We irrigated all the wounds and sutured the laceration of the little finger using 5-0 nylon. We applied artificial dermis to the skin defects under local anesthesia. We administered tetanus immunoglobulin and tetanus toxoid as well as intravenous piperacillin-tazobactam for 3 days, followed by 10 days of intravenous cefazolin sodium.

**Figure 1:**
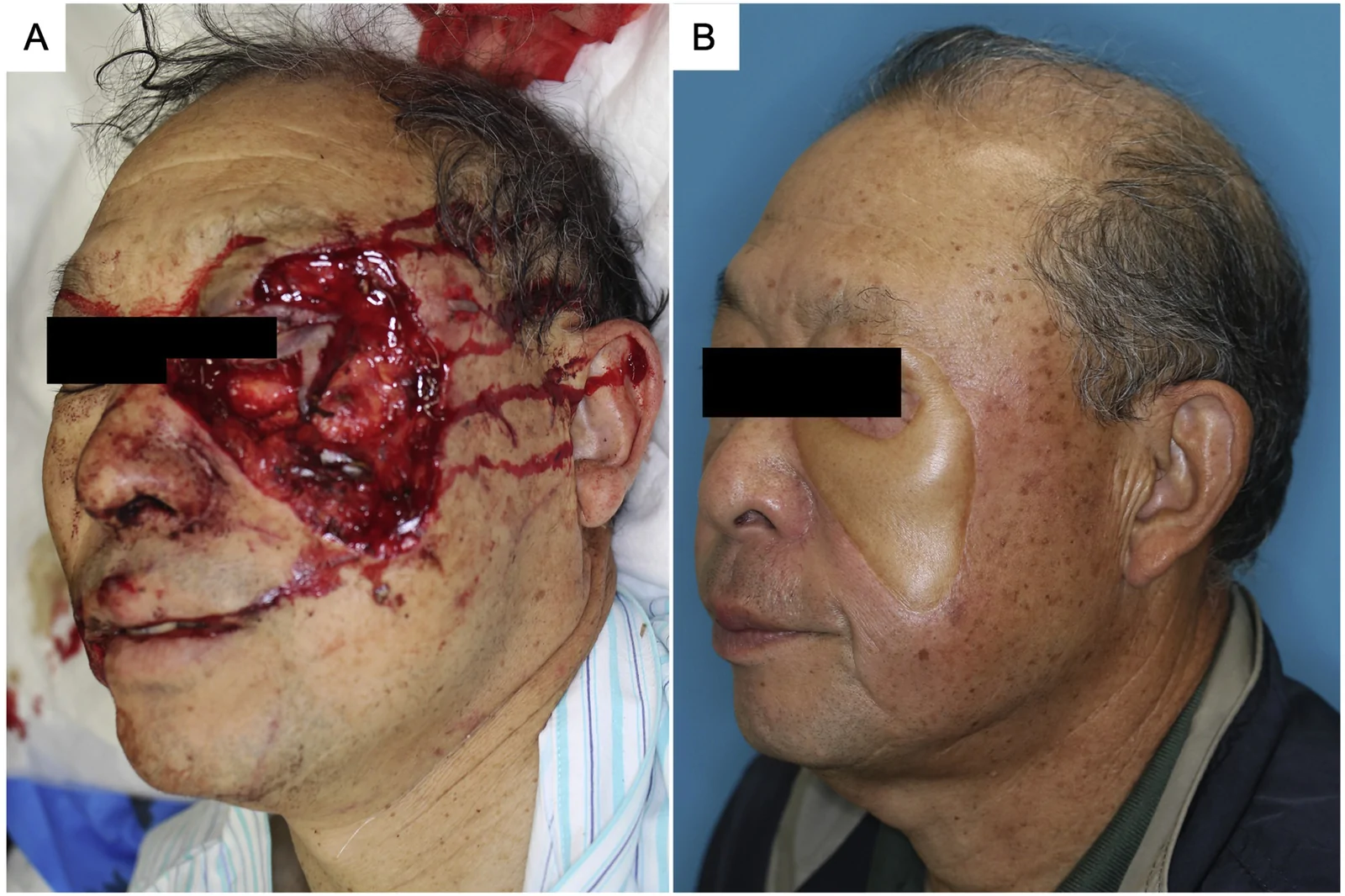
A: Full-thickness tissue loss on the right side of the face. B: 2 years and 8 months after reconstruction using an anterolateral thigh free flap.

On day 10 post-injury, we repaired the right oral commissure with direct closure, and we reconstructed the left eyelid-to-cheek defect using a free anterolateral thigh flap (Fig. 2A-2E). The patient’s postoperative recovery was uneventful, and he was discharged from the hospital on the eleventh postoperative day. Six months after the procedure, he developed ectropion of the left lower eyelid due to the weight of the flap. We resolved his lagophthalmos with flap debulking and fascia lata grafting 8 months after the free-flap surgery. At his most recent follow up, 2 years and 8 months after the initial surgery, there was satisfactory aesthetic and functional recovery (Fig. 1B).

**Figure 2:**
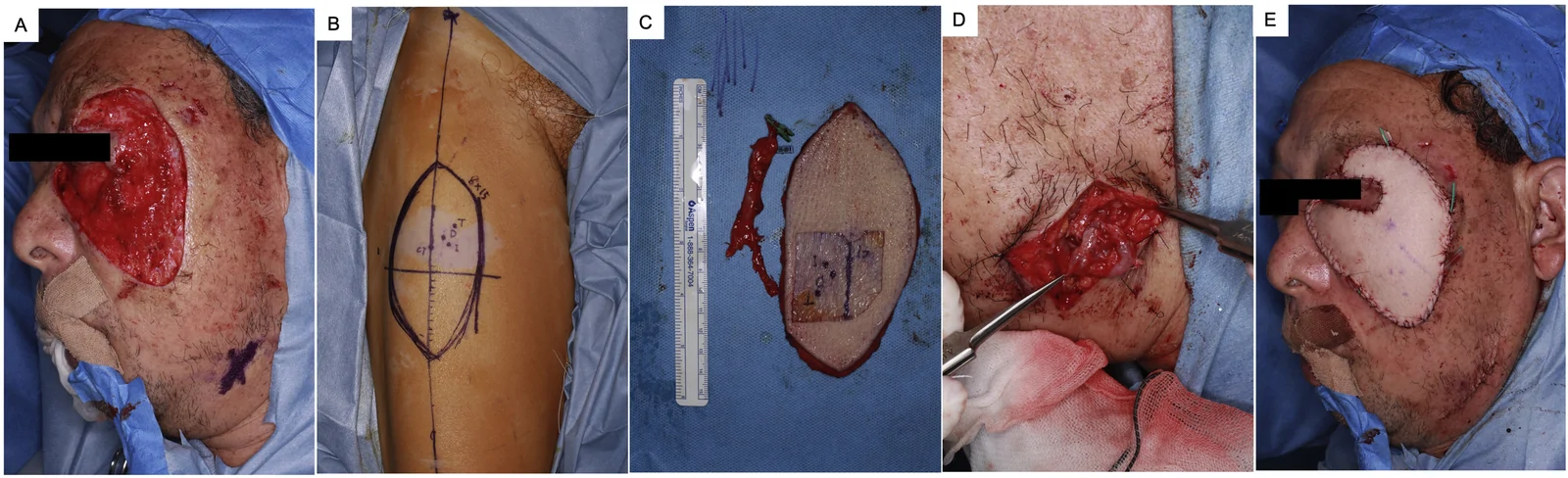
A: Skin defect involving the left periorbital region and cheek. B: Preoperative design of the anterolateral thigh (ALT) flap. C: After elevation of the ALT flap. D: After microvascular anastomosis between the facial artery and vein and the flap pedicle. E: After inset of the flap.

## Discussion

### The eye area is a frequent part of bear injuries

In our series of bear-attack injuries, we noted that the midface—including the eyelid and globe—was most frequently injured. We noted a higher incidence of globe rupture (19%) than previous studies that had reported rates of 4.2% (14/336 patients) to 15% (3/20 patients).^4^^,^^11^ The midface structures—including the globe—seem to be a primary target for bears, according to our results. Once the globe is injured, it is difficult to restore vision. It is important to remind people to protect their faces if they encounter a bear, because eyes are irreplaceable organs for daily activities.

### Key points of Primary management of bear-attack facial injuries

The keys to successful management and treatment of bear-attack injuries to the face are time to treatment, type of injury, and post-traumatic infection. Repair requires a professional approach to achieve optimal functional and cosmetic results. All our patients were transported to our institution on the day of injury, and treatment could be initiated within 24 hours. In addition, all wounds were treated with aggressive debridement and irrigation under general anesthesia, followed by appropriate primary closure. Early intervention for soft tissue injuries is associated with superior postoperative aesthetic outcomes. In contrast, delayed reconstruction could result in progressive soft tissue swelling, loss of anatomical landmarks, and increased technical difficulty in primary closure.^12^

Prophylactic antibiotics were administered to all patients, immunoglobulin preparations were administered to 25 patients, and tetanus toxoid vaccine was administered to 8 patients. As a result, our primary infection rate after the primary procedure was relatively low, at 23% (12% of which involved facial wounds). Even when including donor-site and reconstruction-site infections, the total infection rate was 35% (9 cases), which is comparable to the 34.3% (11/32) reported in a previous study on bear-attack injuries.^13^ Conversely, Kar et al studied 20 patients with facial injuries from bear attack and found no infections after treatment.^11^ Their study, like ours, included patients with fractures, extensive tissue defects, and ocular injuries, and primary closure was performed in 15 of 20 patients.^11^ Previous studies of dog-bite wounds found no significant difference in infection rates between primary closure and secondary healing.^14^^,^^15^ However, the recovery time is shorter and the cosmetic outcome is better with primary closure.^15^ Therefore, especially in areas where cosmetic results are crucial, such as the face, primary closure can be considered a viable option with minimal risk. It is better to perform initial wound-related treatment whenever possible, with appropriate cleansing and antimicrobial administration, than not to perform primary suturing for fear of infection.

### Surgical decision-making process for the treatment of tissue defects

In our case series, patients required up to 4 surgeries during the index hospitalization (Supplementary Figure 1). Nine patients with tissue loss were treated with skin grafts, local skin flaps, or free skin flaps. For non-primary closure of defects, skin grafts, local flaps, and free flaps have been reported as effective treatment options.^16^, ^17^, ^18^ Skin grafting is indicated when adjacent tissue is insufficient. Full-thickness skin grafts are particularly advantageous in facial reconstruction.^16^^,^^19^ Local flaps are associated with superior aesthetic outcomes using adjacent tissue with similar color, texture, and thickness.^16^ Although free flaps may exhibit variability in these characteristics, they remain a preferred option for extensive soft-tissue defects, providing versatile and reliable reconstructive results.^17^

Bear-related facial injuries present unique reconstructive challenges due to complex, multi-site facial wounds. The use of local flaps requires careful design, as the local blood supply may be compromised by the injury. When the tissue defect is extensive and requires substantial volume, free flap reconstruction is an effective option; however, the condition and availability of recipient vessels must be carefully assessed. Furthermore, patient factors, such as advanced age and comorbidities, may limit prolonged surgery. In these situations, less invasive approaches, such as skin grafting, may be more appropriate than free flap reconstruction. We advise clinicians to check the extent of tissue defects when examining patients with facial injury caused by bear attack, and allow appropriate time and plan for appropriate methods of reconstruction. Skin injuries, fractures, and damage to appendages without soft tissue defects may be treated during the initial surgery through suturing, fracture reduction, or lacrimal canalicular repair. If treatment can be completed in a single surgical procedure, it may lead to a shortened hospital stay, potentially facilitating the patient’s early return to society. The severity of facial trauma is frequently assessed using the FISS introduced by Bagheri et al. in 2006.^10^ Higher FISS scores are associated with longer LOS and an increased likelihood of requiring surgical intervention.^20^ However, in our study, no significant correlation was observed between LOS and FISS. This may be attributed to the predominantly low FISS scores in our cohort, with a median of 2.00 [IQR, 1.00–3.00]. Although the FISS evaluates injury based on fracture sites and cutaneous laceration length, it does not account for soft tissue defects. Because our findings suggest that soft tissue defects may also influence LOS, a more comprehensive assessment may be warranted. Of course, large wounds take time to heal, and there may be issues with scarring. Clinicians need to follow up with patients and may need to make minor corrections, as noted in our representative cases, depending on the patient's cosmetic and functional results.

### Limitations

First, we only reviewed patients who survived to present to the Iwate Medical University Hospital, and this review involved a limited number of patients. Details of some minor complications could not be analyzed because of the retrospective nature of our study. Second, although GLM analysis was performed to adjust for potential confounding variables, such as FISS scores, infection, and soft tissue defects, it should be considered strictly exploratory due to the limited sample size (n = 26) and the risk of statistical overfitting. Consequently, definitive conclusions regarding independent prognostic factors could not be drawn. Future multicenter studies with larger cohorts are required to perform robust multivariable analyses. Third, bear attacks typically cause severe, highly comminuted facial fractures that do not conform to standard fracture patterns, making objective scoring using FISS criteria challenging. Future prospective studies focusing on bear-inflicted trauma are warranted to establish validated severity assessment protocols. Finally, the LOS may be influenced by regional differences in discharge criteria and health insurance systems. Long-term post-discharge outcomes or disease-free metrics could not be assessed because some patients received follow-up and secondary care at local hospitals near their residences after primary treatment.

## Conclusion

Bear trauma is characterized by a high incidence of periorbital damage, which is difficult to remedy with current medical care. Soft tissue defects might necessitate multiple surgeries. We believe that a basic approach using aggressive irrigation and debridement, as well as primary closure (when possible) under general anesthesia, is optimal for facial injuries resulting from bear attacks.

## Ethics approval

The institutional review board granted approval for this study (MH2024-017), and patients were able to opt out of consent for research participation. Written informed consent was obtained from the patients for the publication of any identifying photographs.

## Declaration of competing interest

None declared.
