# Supplementary material for: Facial injuries from bear attacks: A retrospective analysis highlighting periorbital trauma and the need for tissue defect management
Source: JPRAS Open. 2026 Sep 30;52:618–25. doi: 10.1016/j.jpra.2026.09.029 (PMC13682383; doi:10.1016/j.jpra.2026.09.029)
Supplement: Supplementary file 1 [file mmc2.docx]

Supplementary Figure 1. Distribution of Clinical Characteristics in the Patient Cohort.

Histograms showing the distributions of age, length of hospital stay (LOS), Facial Injury Severity Scale (FISS) score, antibiotic duration, and the number of surgeries among the study cohort (n=26).
