# Supplementary material for: Facial injuries from bear attacks: A retrospective analysis highlighting periorbital trauma and the need for tissue defect management
Source: JPRAS Open. 2026 Sep 30;52:618–25. doi: 10.1016/j.jpra.2026.09.029 (PMC13682383; doi:10.1016/j.jpra.2026.09.029)
Supplement: Supplementary file 2 [file mmc3.docx]

Supplementary Table 1. Detailed facial bone fractures and corresponding maxillofacial management.

| Case | Age / Sex | Fracture Sites | Management by Site |
| --- | --- | --- | --- |
| 1 | 60 / M | Nasal, Zygoma | Nasal: CR/ Zygoma: Conservative |
| 2 | 75 / M | Mandible (Subcondylar) | Mandible: IMF → ORIF + IMF |
| 3 | 70 / M | Zygoma | Zygoma: ORIF |
| 4 | 77 / M | Nasal | Nasal: CR |
| 5 | 62 / M | — | — |
| 6 | 71 / F | Zygoma, Mandible (Subcondylar) | Zygoma: OR/ Mandible: None → ORIF + IMF |
| 7 | 74 / M | Zygoma, Orbital wall, Nasal-Ethmoid, Maxilla | Zygoma: ORIF/ Other sites: Conservative |
| 8 | 67 / M | Frontal, Zygoma | Frontal, Zygoma: ORIF |
| 9 | 69 / M | Nasal-Ethmoid, Zygoma, Maxilla | Nasal-Ethmoid: CR/ Zygoma, Maxilla: ORIF |
| 10 | 69 / M | Nasal, Maxilla | Nasal: CR/ Maxilla: ORIF |
| 11 | 67 / F | — | — |
| 12 | 83 / M | Frontal, Zygoma | Frontal, Zygoma: ORIF |
| 13 | 64 / M | — | — |
| 14 | 66 / M | — | — |
| 15 | 45 / M | Nasal | Nasal: CR |
| 16 | 82 / M | — | — |
| 17 | 52 / M | — | — |
| 18 | 81 / M | Nasal-Ethmoid, Zygoma, Maxilla | Nasal-Ethmoid, Zygoma, Maxilla: ORIF |
| 19 | 72 / F | Nasal-Ethmoid, Maxilla | Nasal-Ethmoid, Maxilla: Conservative |
| 20 | 79 / F | — | — |
| 21 | 81 / M | Frontal | Frontal: Conservative |
| 22 | 71 / M | Nasal-Ethmoid | Nasal-Ethmoid: Conservative |
| 23 | 70 / M | Nasal, Maxilla | Nasal: CR/ Maxilla: ORIF |
| 24 | 65 / M | — | — |
| 25 | 79 / M | — | — |
| 26 | 72 / M | — | — |

Abbreviations: ORIF, Open Reduction and Internal Fixation; Frontal, Frontal sinus fracture; CR, Closed reduction; IMF, Intermandibular fixation; —, None.

Supplementary Table 2. Length of hospital stay, number of surgeries, and infections during initial hospitalization.

| Case | Age /Sex | Surgeries  (No.) | LOS (days) | Primary Infection | Reconstructive / Donor Site Infection |
| --- | --- | --- | --- | --- | --- |
| 1 | 60/M | 1 | 19 | — | — |
| 2 | 75/M | 2 | 30 | — | — |
| 3 | 70/M | 1 | 15 | — | — |
| 4 | 77/M | 1 | 20 | Head | — |
| 5 | 62/M | 1 | 14 | Hand | — |
| 6 | 71/F | 2 | 28 | Forearm | — |
| 7 | 74/M | 2 | 35 | — | Thigh donor site |
| 8 | 67/M | 4 | 49 |  | Face (ALT Flap) |
| 9 | 69/M | 1 | 8 | — | — |
| 10 | 69/M | 1 | 11 | — | — |
| 11 | 67/F | 2 | 30 | — | — |
| 12 | 83/M | 1 | 15 | — | — |
| 13 | 64/M | 1 | 6 | — | — |
| 14 | 66/M | 1 | 6 | — | — |
| 15 | 45/M | 1 | 10 | — | — |
| 16 | 82/M | 1 | 8 | Head | — |
| 17 | 52/M | 1 | 6 | — | — |
| 18 | 81/M | 1 | 22 | Face (Cheek) | — |
| 19 | 72/F | 3 | 48 | — | — |
| 20 | 79/F | 1 | 14 | — | — |
| 21 | 81/M | 3 | 51 | — | Face (Nasal flap) |
| 22 | 71/M | 1 | 9 | — | — |
| 23 | 70/M | 1 | 11 | — | — |
| 24 | 65/M | 2 | 21 | — | — |
| 25 | 79/M | 2 | 29 | — | — |
| 26 | 72/M | 1 | 8 | Thigh | — |

Surgeries(No.) refers to the number of surgeries performed during initial hospitalization. Primary infection refers to infection originating from the initial traumatic injury site, regardless of the time of onset during hospitalization. Abbreviations: LOS, length of hospital stay; —, none; ALT, anterolateral thigh.
