# Supplementary material for: Facial injuries from bear attacks: A retrospective analysis highlighting periorbital trauma and the need for tissue defect management
Source: JPRAS Open. 2026 Sep 30;52:618–25. doi: 10.1016/j.jpra.2026.09.029 (PMC13682383; doi:10.1016/j.jpra.2026.09.029)
Supplement: Supplementary file 3 [file mmc1.doc]

STROBE Statement—Checklist of items that should be included in reports of ***cohort studies***

|  | Item No | Recommendation |
| --- | --- | --- |
| **Title and abstract** | 1 | (*a*)Indicate the study’s design with a commonly used term in the title or the abstract  Author response: The study design (e.g., retrospective design) is clearly presented in the opening sentence of the Methods section. |
|  |  | (*b*) Provide in the abstract an informative and balanced summary of what was done and what was found  Author response: The abstract provides a concise, informative, and balanced summary of the study methods and main findings, in accordance with the journal's author guidelines. |
| Introduction | | |
| Background/rationale | 2 | Explain the scientific background and rationale for the investigation being reported  Author response: The scientific background and rationale for conducting this study are clearly explained in the introduction. |
| Objectives | 3 | State specific objectives, including any prespecified hypotheses  Author response: The specific objectives are clearly stated in the introduction section. |
| Methods | | |
| Study design | 4 | Present key elements of study design early in the paper  Author response: The study design (retrospective design) is clearly presented in the opening sentence of the Methods section. |
| Setting | 5 | Describe the setting, locations, and relevant dates, including periods of recruitment, exposure, follow-up, and data collection  Author response: The setting, including locations, time frame, and data collection, is described in detail in the Methods section. |
| Participants | 6 | (a) Give the eligibility criteria, and the sources and methods of selection of participants. Describe methods of follow-up  Author response: Eligibility criteria, sources, methods of participant selection, and follow-up procedures are clearly described in the Methods section. |
|  |  | (*b*) For matched studies, give matching criteria and number of exposed and unexposed  Author response: Not applicable, as the study does not use a matched design. |
| Variables | 7 | Clearly define all outcomes, exposures, predictors, potential confounders, and effect modifiers. Give diagnostic criteria, if applicable  Author response: All outcomes are clearly defined in the Methods section, and diagnoses were made by board-certified plastic surgeons where applicable. |
| Data sources/ measurement | 8* | For each variable of interest, give sources of data and details of methods of assessment (measurement). Describe comparability of assessment methods if there is more than one group  Author response: For each variable of interest, the source of data and method of assessment are described in the Methods section. When applicable, comparability of assessment methods across groups is addressed. |
| Bias | 9 | Describe any efforts to address potential sources of bias  Author response: Due to the retrospective study, direct bias control was limited. However, potential sources of bias are discussed in the Limitations section. |
| Study size | 10 | Explain how the study size was arrived at  Author response: As this is a retrospective study, the study size was determined based on the available data and is explained in the Methods section. |
| Quantitative variables | 11 | Explain how quantitative variables were handled in the analyses. If applicable, describe which groupings were chosen and why  Author response: The handling of quantitative variables is described in the Methods section. |
| Statistical methods | 12 | (a) Describe all statistical methods, including those used to control for confounding  Author response: Statistical methods used in the analysis are described in the Methods section. No adjustment for confounding factors was performed, as it was not applicable to the study design and objectives. |
|  |  | (*b*) Describe any methods used to examine subgroups and interactions  Author response: Any methods used to examine subgroups and interactions are described in the Methods section. |
|  |  | (*c*) Explain how missing data were addressed  Author response: For score calculations, missing sub-items were omitted, and total FISS scores were calculated based on the available items. For overall analyses, there were no missing data across the variables evaluated. |
|  |  | (*d*) If applicable, explain how loss to follow-up was addressed  Author response: Not applicable due to the retrospective design of the study. |
|  |  | (*e*) Describe any sensitivity analyses  Author response: Sensitivity analyses were not conducted, as this was a retrospective study. |
| Results | | |
| Participants | 13* | (a) Report numbers of individuals at each stage of study—eg numbers potentially eligible, examined for eligibility, confirmed eligible, included in the study, completing follow-up, and analysed  Author response: The number of individuals at each stage of the study is reported in the Methods and Results sections. |
|  |  | (b) Give reasons for non-participation at each stage  Author response: Not applicable, as the study is retrospective and no active recruitment or consent process was required. |
|  |  | (c) Consider use of a flow diagram  Author response: A flow diagram was not created due to the retrospective nature of the study. |
| Descriptive data | 14* | (a) Give characteristics of study participants (eg demographic, clinical, social) and information on exposures and potential confounders  Author response: The characteristics and information on exposures and potential confounders are reported in the Results section, as appropriate. |
|  |  | (b) Indicate number of participants with missing data for each variable of interest  Author response: For one case, data on scar length required for calculating the FISS score were missing, and the score was evaluated using the remaining available items. For all other primary variables across the 26 analyzed cases, there were no missing data. |
|  |  | (c) Summarise follow-up time (eg, average and total amount)  Author response: Follow-up time is not applicable in this retrospective analysis. The observation period in this study was defined as the duration of the initial hospitalization. |
| Outcome data | 15* | Report numbers of outcome events or summary measures over time  Author response: Outcome events and summary measures are reported in the Results section. |
| Main results | 16 | *(*a) Give unadjusted estimates and, if applicable, confounder-adjusted estimates and their precision (eg, 95% confidence interval). Make clear which confounders were adjusted for and why they were included  Author response: Continuous variables were summarized as median [IQR]. Group comparisons used the Mann–Whitney U test with p < 0.05 as the threshold for significance. Generalized Linear Model (GLM) with a gamma distribution and log link function was used to estimate adjusted effects and their 95% confidence intervals, accounting for the right-skewed length of hospital stay (LOS). Adjustments were made for potential confounders, including the FISS score, soft tissue defects, and infection, to evaluate their independent associations with LOS. |
|  |  | (*b*) Report category boundaries when continuous variables were categorized  Author response: Continuous variables were not categorized; they were analysed and presented as continuous data using the median [IQR]. |
|  |  | (*c*) If relevant, consider translating estimates of relative risk into absolute risk for a meaningful time period  Author response: This was not applicable, as relative risk estimates were not calculated in this study. |
| Other analyses | 17 | Report other analyses done—eg analyses of subgroups and interactions, and sensitivity analyses  Author response: Any subgroup or additional analyses performed are reported in the Results section. |
| Discussion | | |
| Key results | 18 | Summarise key results with reference to study objectives  Author response: The key results are summarized in the Discussion section with reference to the study objectives. |
| Limitations | 19 | Discuss limitations of the study, taking into account sources of potential bias or imprecision. Discuss both direction and magnitude of any potential bias  Author response: Study limitations, including potential sources and magnitude of bias or imprecision, are thoroughly discussed in the Limitations section. |
| Interpretation | 20 | Give a cautious overall interpretation of results considering objectives, limitations, multiplicity of analyses, results from similar studies, and other relevant evidence  Author response: The Discussion section provides a cautious overall interpretation of the findings, considering study objectives, limitations, multiple analyses, related studies, and other relevant evidence. |
| Generalisability | 21 | Discuss the generalisability (external validity) of the study results  Author response: The generalisability (external validity) of the study findings is discussed in the Limitations portion of the Discussion section. |
| Other information | | |
| Funding | 22 | Give the source of funding and the role of the funders for the present study and, if applicable, for the original study on which the present article is based  Author response: The source of funding and the role of the funders in this study are disclosed in the Funding section. |

*Give information separately for exposed and unexposed groups.

**Note:** An Explanation and Elaboration article discusses each checklist item and gives methodological background and published examples of transparent reporting. The STROBE checklist is best used in conjunction with this article (freely available on the Web sites of PLoS Medicine at http://www.plosmedicine.org/, Annals of Internal Medicine at http://www.annals.org/, and Epidemiology at http://www.epidem.com/). Information on the STROBE Initiative is available at http://www.strobe-statement.org.
